# A Case of Acute Myeloid Leukemia with a Previously Unreported Translocation (14; 15) (q32; q13)

**DOI:** 10.1155/2014/921240

**Published:** 2014-11-11

**Authors:** Mohamad Khawandanah, Bradley Gehrs, Shibo Li, Jennifer Holter Chakrabarty, Mohamad Cherry

**Affiliations:** ^1^Hematology-Oncology Section, Department of Medicine, The University of Oklahoma Health Sciences Center, Oklahoma City, OK 73104, USA; ^2^University of Oklahoma Health Sciences Center, Stephenson Cancer Center, 800 NE 10th Street, Oklahoma City, OK 73102, USA; ^3^Department of Pathology, The University of Oklahoma Health Sciences Center, Oklahoma City, OK 73104, USA; ^4^Department of Pediatrics, The University of Oklahoma Health Sciences Center, Oklahoma City, OK 73104, USA

## Abstract

*Background*. We hereby describe what we believe to be the first reported case of t (14; 15) (q32; q13) associated with acute myeloid leukemia (AML). *Methods*. PubMed, Embase, and OVID search engines were used to review the related literature and similar published cases. *Case*. A47-year-old female presented in December 2011 with AML (acute myelomonocytic leukemia) with normal cytogenetics; molecular testing revealed FLT-3 internal tandem duplication (ITD) mutation, while no mutations involving FLT3 D385/I836, NPM1 exon 12, or KIT exons 8 and 17 were detected. She was induced with 7 + 3 (cytarabine + idarubicin) and achieved complete remission after a second induction with high-dose cytarabine (HiDAC) followed by uneventful consolidation. She presented 19 months after diagnosis with relapsed disease. Of note, at relapse cytogenetic analysis revealed t (14; 15) (q32; q13), while FLT-3 analysis showed a codon D835 mutation (no ITD mutation was detected). She proved refractory to the initial clofarabine-based regimen, so FLAG-idarubicin then was used. She continued to have persistent disease, and she was discharged on best supportive care. *Conclusion*. Based on this single case of AML with t (14; 15) (q32; q13), this newly reported translocation may be associated with refractory disease.

## 1. Introduction

Genetic evaluation plays an integral role in the classification of AML [1–3]. Approximately 50% of patients with de novo AML have cytogenetics abnormalities. As reflected in the latest WHO classification scheme for AML, certain cytogenetic abnormalities are used to diagnose patients with AML regardless of the blast count. Cytogenetic analysis also identifies abnormalities with prognostic and therapeutic implications, it can aid in monitoring the therapeutic response, and in the future it likely will have a greater role in abnormality-tailored approaches.

Similarly, mutational analysis plays an important and expanding role in the diagnosis and management of AML. FLT3 is a member of the class III receptor tyrosine kinase family important for the normal development of hematopoietic stem cells and the immune system. FLT3 is the most commonly mutated gene in AML, with mutations occurring in about 30% of cases overall. There are several known activating mutations of FLT3 in AML, including internal tandem duplications (ITD) and mutations in the activation loop of the second tyrosine kinase domain (TKD) such as* FLT-3 D835/I836*. FLT3-ITD mutations are an adverse prognostic factor. However, the significance of FLT3-TKD mutations is unclear; they are overrepresented in the PML-RARA and inv(16) AML subtypes and more generally in the intermediate-risk cytogenetic subgroup [2].

We hereby describe what we believe to be the first reported case of t (14; 15) (q32; q13) associated with AML. Relapse in this case also is notable for alterations in the FLT3 status (isolated FLT3-ITD mutation at diagnosis; isolated FLT3 D835 mutation at relapse) in addition to the appearance of this newly described translocation.

## 2. Case Presentation

The patient was a 47-year-old white female who initially presented with abdominal pain. She was noted to have leukocytosis with numerous blasts in her peripheral blood; her CBC included a WBC count of 16,900/mm^3^ (with a differential of 56% blasts, 1% promyelocytes, 1% myelocytes, 1% metamyelocytes, 1% bands, 2% neutrophils, 30% lymphocytes, and 8% monocytes), a hemoglobin of 7.5 g/dL, and a platelet count of 43,000/mm^3^. She did not have a significant medical or surgical history, although she had a 27-pack-year smoking history. Her father died from pancreatic cancer. On exam her vital signs were within normal limits. No splenomegaly or lymphadenopathy was noted.

Bone marrow specimens were obtained for morphologic, immunophenotypic, and genetic analyses. Based on morphology and flow cytometric analysis, the patient was diagnosed with AML (acute myelomonocytic leukemia). Flow cytometry showed 25% blasts expressing CD45 (moderate intensity), CD34, CD13, CD117 (partial), CD33 (partial), HLA-DR (partial), CD4 (partial), and CD15 (partial); the blasts had no significant expression of CD14, CD64, CD10, CD19, CD20, surface immunoglobulin light chains, CD2, CD3, CD5, CD7, CD8, or CD56. It also noted 31% monocytes expressing CD45 (bright), CD13, CD33, CD4, CD64, HLA-DR, CD14 (partial), and CD15 (partial) with possible slight expression of CD34 and CD2; the monocytes exhibited no expression of CD117 or the other analyzed lymphoid antigens.

Routine cytogenetic analysis revealed a normal female karyotype (46, XX [21]). A multiplex, nested reverse transcription PCR assay for 16 of the more common recurrent chromosomal rearrangements associated with acute leukemia did not detect any abnormalities. Molecular assays for detection of mutations involving FLT3 ITD, FLT3 D835/I836, NPM1 exon 12, and KIT exons 8 and 17 were performed; only a FLT3 ITD mutation was detected.

The patient was started on induction chemotherapy with 7 + 3 (cytarabine 100 mg/m^2^/day continuous IV infusion on days 1–7 and idarubicin 12 mg/m^2^/d IV on days 1–3). The day 18 bone marrow evaluation was suspicious for residual AML, so a second induction was completed with HiDAC (cytarabine 3 g/m^2^ IV q12 hour × 6 doses on days 1, 3, and 5). A bone marrow evaluation after the second induction revealed no evidence of residual AML. She was consolidated with 4 additional cycles of HiDAC. She achieved complete remission with a normal CBC and transfusion independence. She was not a candidate for allogeneic stem cell transplantation due to the lack of an appropriate donor and concerning psychosocial assessment.

Fourteen months later, she presented with relapsed AML. Her CBC showed a WBC count of 4,000/mm^3^ (53% blasts, 1% bands, 4% neutrophils, 41% lymphocytes, and 1% monocytes), a hemoglobin of 11.0 g/dL, and a platelet count of 83,000/mm^3^.

A restaging bone marrow confirmed relapsed AML. Flow cytometry showed somewhat similar results to those at diagnosis. There were 36% blasts expressing CD45 (dim), CD13, CD117 (partial), CD33, CD4 (partial), and CD15 (partial); the blasts exhibited minimal expression of CD34 and HLA-DR and no significant expression of CD14, CD64, CD10, CD19, CD20, surface immunoglobulin light chains, CD2, CD3, CD5, CD7, CD8, or CD56. There also were 25% monocytes expressing CD45 (bright), CD13 (partial), CD33, CD4, CD64, HLA-DR (partial), CD14 (partial), and CD15; the monocytes exhibited no expression of CD34, CD117, or the analyzed lymphoid antigens.

**Figure 1:**
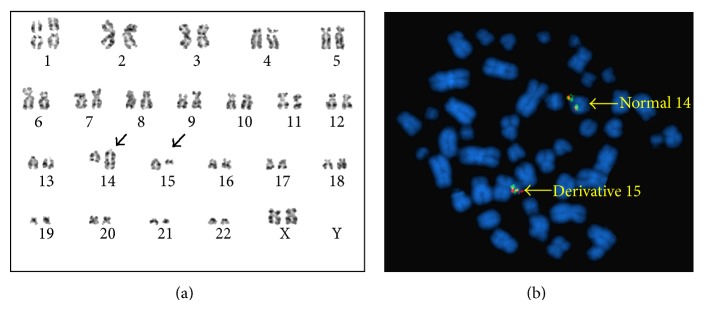
(a) G-banded chromosome analysis showed an apparently balanced translocation between chromosomes 14 and 15 at breakpoints of 14q32 and 15q13, respectively. (b) FISH analysis using IGHG1 break-apart probe revealed intact signals on a normal chromosome 14 and a derivative chromosome 15.

Repeated routine cytogenetic analysis revealed an abnormal karyotype with t (14; 15) (q32; q13) in 16 of 20 analyzed cells (Figure 1). Molecular studies no longer showed a FLT-3 ITD mutation, but a FLT-3 D835 mutation was detected (Table 1).

Her relapse initially was treated with a clofarabine-based regimen (clofarabine 30 mg/m^2^/day IV on days 1–5, cytarabine 20 mg/m^2^/day SC on days 1–14, and sorafenib 400 mg PO BID on days 1–14). Unfortunately the postinduction day 22 bone marrow showed persistent AML, including the presence of t (14; 15) (q32; q13). A second induction was performed with FLAG-idarubicin (fludarabine 30 mg/m^2^/day IV on days 1–5, cytarabine 3000 mg/m^2^/day IV on days 1–5, and idarubicin 10 mg/m^2^/day IV on days 1–3); this treatment was complicated with prolonged neutropenia and a disseminated Fusarium infection involving sinus and cutaneous tissues. The day 40 bone marrow biopsy was consistent with persistent AML; it was hypocellular with left-shifted granulocytic maturation, decreased erythropoiesis, and dysmegakaryopoiesis. As a result, the patient was discharged on hospice care.

## 3. Discussion

To the best of our knowledge, this is the first described case of AML with t (14; 15) (q32; q13). The genes activated or inactivated as a result of this translocation as well as the overall significance of this translocation in the disease process are unclear. Chromosome 14q32 encodes the IgH gene, which produces heavy chain immunoglobulin. Translocations involving the IgH gene/14q32 are well established in multiple hematologic malignancies including diffuse large B-cell lymphoma [3], mantle cell lymphoma [4], precursor T cell acute lymphoblastic leukemia/lymphoma [5], chronic lymphocytic leukemia [6], and multiple myeloma [7]. Another gene located at chromosome 14q32 is BCL11B (B-cell lymphoma/leukemia 11B), which plays a critical role in T-cell differentiation and proliferation and typically is associated with T-lymphoid malignancies. BCL11B encodes a C2H2-type zinc finger protein and is closely related to BCL11A, a gene whose translocation may be associated with B-cell malignancies [8]. BCL11B was first associated with hematological malignancies due to its recurrent involvement along with the homeobox transcription factor TLX3 (previously HOX11L2) in a significant percentage of pediatric T-cell acute lymphoblastic leukemia (T-ALL) cases carrying the cryptic t (5; 14) (q35; q32) [9]. Interestingly, in 2004 Bezrookove et al. [10] described an unusual case of AML with t (6; 14) (q25–q27; q32) affecting the BCL11B gene. In 2011 Oliveira et al. described a case of a child with bilineal T/myeloid acute leukemia associated with del (9q) (q13q22) and TLX3/BCL11B fusion due to the cryptic t (5; 14) (q35; 32) [11]. More recently Ahmad et al. [12] suggested that BCL11B could be a potential oncogene involved in AML with 14q32 aberrations; the identified 4 cases of AML with BCL11B rearrangements (each with a different partner chromosome) expressed both myeloid and T-cell markers and carried AML-associated FLT3 internal tandem duplications (one case also had a FLT-3 D835 mutation). Given the absence of pan-T-cell markers in our case, potential involvement by BCL11B certainly is only conjecture.

Additional sporadic cases of AML with translocations involving 14q32 have been reported. For example, Tecimer et al. [13] reported a case of AML (AML-M0) with t (1; 14) (p13; q32). Ahmad et al. [12] reported a case of AML (AML-M0/M1) with t (14; 17) (q32; q11.2).

In our case the relapsed leukemia demonstrated refractoriness to multiagent chemotherapy regimens. This may represent either clonal evolution or an entirely new clone which can reflect a therapy related resistant disease after exposure to leukemia treatment on initial presentation. The lack of the FLT-3 ITD mutation, the presence of a FLT-3 D835 codon mutation, and the new onset of t (14; 15) (q32; q13) could support the second interpretation. In the setting of AML, this translocation is of unknown potential but may be linked to refractoriness to chemotherapy. However, potential unidentified mutation involving partner genes can lead also refractory disease. Collecting and reporting data of rare chromosomal abnormalities will potentially add more information concerning the pathogenesis and prognosis of AML. Additionally, it may translate to improved patient outcome and management in the future if identified earlier in the clinical course which may aid in selecting patients to undergo more aggressive chemotherapy regimens and allogeneic stem cell transplant.

## Figures and Tables

**Table 1:** Molecular and cytogenetic test results at initial diagnosis and at time of relapse.

Test	Result at diagnosis	Repeated result at time of relapse
FLT-3 ITD	Detected	Not detected
FLT-3 D835	Not detected	Detected
FLT-3 I836	Not detected	Not detected
NPM1 exon 12	Not detected	Not performed
Cytogenetics	46, XX [21]	46, XX, t (14; 15) (q32; q13) [16]/46, XX [4]

## References

[1] Gary Gilliland D., Griffin J. D. (2002). The roles of FLT3 in hematopoiesis and leukemia. *Blood*.

[2] Kok C. H., Brown A. L., Perugini M., Iarossi D. G., Lewis I. D., D'Andrea R. J. (2013). The preferential occurrence of FLT3-TKD mutations in inv(16) AML and impact on survival outcome: a combined analysis of 1053 core-binding factor AML patients. *British Journal of Haematology*.

[3] Cigudosa J. C., Parsa N. Z., Louie D. C. (1999). Cytogenetic analysis of 363 consecutively ascertained diffuse large B-cell lymphomas. *Genes Chromosomes Cancer*.

[4] Harris N. L., Jaffe E. S., Stein H., Banks P. M., Chan J. K. C., Cleary M. L., Delsol G., De Wolf-Peeters C., Falini B., Gatter K. C., Grogan T. M., Isaacson P. G., Knowles D. M., Mason D. Y., Muller-Hermelink H.-K., Pileri S. A., Piris M. A., Ralfkiaer E., Warnke R. A. (1994). A revised European-American classification of lymphoid neoplasms: a proposal from the International Lymphoma Study Group. *Blood*.

[5] Heerema N. A., Sather H. N., Sensel M. G. (1998). Frequency and clinical significance of cytogenetic abnormalities in pediatric T-lineage acute lymphoblastic leukemia: a report from the Children's Cancer Group. *Journal of Clinical Oncology*.

[6] Hanada M., Delia D., Aiello A., Stadtmauer E., Reed J. C. (1993). bcl-2 Gene hypomethylation and high-level expression in B-cell chronic lymphocytic leukemia. *Blood*.

[7] Avet-Loiseau H., Facon T., Grosbois B., Magrangeas F., Rapp M.-J., Harousseau J.-L., Minvielle S., Bataille R. (2002). Oncogenesis of multiple myeloma: 14q32 and 13q chromosomal abnormalities are not randomly distributed, but correlate with natural history, immunological features, and clinical presentation. *Blood*.

[8] Gao Y., Wu H., He D. (2013). Downregulation of BCL11A by siRNA induces apoptosis in B lymphoma cell lines. *Biomedical Reports*.

[9] Bernard O. A., Busson-LeConiat M., Ballerini P., Mauchauffé M., Della Valle V., Monni R., Khac F. N., Mercher T., Penard-Lacronique V., Pasturaud P., Gressin L., Heilig R., Daniel M.-T., Lessard M., Berger R. (2001). A new recurrent and specific cryptic translocation, t(5;14)(q35;q32), is associated with expression of the Hox11L2 gene in T acute lymphoblastic leukemia. *Leukemia*.

[10] Bezrookove V., van Zelderen-Bhola S. L., Brink A., Szuhai K., Raap A. K., Barge R., Beverstock G. C., Rosenberg C. (2004). A novel t(6;14)(q25~q27;q32) in acute myelocytic leukemia involves the BCL11B gene. *Cancer Genetics and Cytogenetics*.

[11] Oliveira J. L., Kumar R., Khan S. P. (2011). Successful treatment of a child with T/myeloid acute bilineal leukemia associated with *TLX3/BCL11B* fusion and 9q deletion. *Pediatric Blood & Cancer*.

[12] Ahmad F., Dalvi R., Mandava S., Das B. R. (2007). Acute Myelogeneous Leukemia (M0/M1) with novel chromosomal abnormality of t(14;17) (q32; q11.2). *The American Journal of Hematology*.

[13] Tecimer C., Loy B. A., Martin A. W. (1999). Acute myeloblastic leukemia (M0) with an unusual chromosomal abnormality: Translocation (1;14)(p13;q32). *Cancer Genetics and Cytogenetics*.

